# PTEN: Regulation, Signalling and Targeting in Cancer

**DOI:** 10.3390/cancers18091320

**Published:** 2026-04-22

**Authors:** Larissa Kotelevets, Mark G. H. Scott, Eric Chastre

**Affiliations:** 1 Sorbonne Université, INSERM, Centre de Recherche Saint-Antoine (CRSA), UMR_S938, 75012 Paris, France; 2 Université Paris Cité, INSERM U1016, CNRS UMR8104, Institut Cochin, 75014 Paris, France

PTEN (phosphatase and tensin homolog deleted on chromosome ten)/MMAC1 (mutated in multiple advanced cancers) was discovered by two groups in 1997 as a novel tumor suppressor gene located at chromosome 10q23 [1,2]. Concurrently, it was identified and designated TEP-1 (TGF-β-regulated and epithelial cell-enriched phosphatase) by a third group in search of novel dual-specificity phosphatases [3]. PTEN’s role as a critical gatekeeper of neoplastic progression rapidly emerged due to its inactivation in a broad range of cancers. PTEN is now considered one of the most frequently affected tumor suppressors in human carcinogenesis. More than 24,000 original articles have been published on PTEN since its discovery (PubMed Central, April 2026). In addition to somatic inactivation, heterozygous germline PTEN mutations cause autosomal dominant syndromes, associated with hamartomas (PTEN hamartoma tumor syndrome, PHTS), increased susceptibility to cancers, and neurodevelopmental alterations. This highlights the role of PTEN in both normal development and tissue maintenance. Accordingly, PTEN restrains cell proliferation, favors cell differentiation, regulates cell metabolism, triggers apoptosis and inhibits cell migration and invasiveness. These pleiotropic actions rely on its phosphatase activity. PTEN dephosphorylates Ser/Thr and Tyr amino-acid residues in protein substrates, as well as the phosphatidylinositol (3,4,5)-trisphosphate generated by phosphatidylinositol 3-kinase (PI3K), thereby curtailing the PI3K/AKT signaling pathway. It also performs phosphatase-independent activities in the cytoplasm and nucleus to restrain cell migration, stabilize the tumor suppressor p53, promote DNA damage repair, and sustain chromosome integrity. Even a slight decrease in PTEN cellular levels can cause cancer development and progression in a tissue-dependent manner [4]. Thus, investigating the fine-tuning of PTEN expression and biological activities represents an important area of research. PTEN is regulated at multiple different levels through epigenetic processes, transcription, RNA stability/degradation, post-translational modifications, recruitment into molecular scaffolding complexes, and subcellular targeting [5].

In this context, this Special Issue aimed to display and discuss different levels of PTEN regulation and function, the biological impact associated with its dysregulation, the definitions of biomarkers with prognostic value, and the identification of putative targets for precision medicine.

With regard to PTEN accumulation, Glena Travis et al. focus on PTEN regulation at transcriptional and post-transcriptional levels [6]. In this review article, the authors detail the tissue selectivity of PTEN inactivation, as well as the relative importance of gene mutation, deletion, and promoter methylation, in relation to protein levels. They emphasize PTEN post-transcriptional regulation by antagonistic networks of microRNAs and competing endogenous RNAs (ceRNAs). Special attention is given to PTEN pseudogenes (PTENP1 sense, and antisenses α and β). PTENP1-ASα silences the PTEN promoter, whereas PTENP1-ASβ favors PTENP1-S nuclear export that acts as a decoy sponging miRNAs targeting PTEN. The mRNA levels of PTEN and PTENP1-S are correlated, and the downmodulation of PTENP1 in cancer due to copy number loss and promoter methylation makes it a potential tumor suppressor. Thus, long non-coding RNAs (lncRNAs) and microRNAs controlling PTEN mRNA levels are levers that can be harnessed in some PTEN-related malignancies.

In their review, Ana González-García et al. focus on another level of PTEN regulation, i.e., post-translational modifications (PTMs) [7]. PTEN is subject to numerous modifications, including phosphorylation of Ser/Thr and Tyr amino-acid residues, acetylation, oxidation, ubiquitination, and SUMOylation. Depending on the residues and type of modification, the activity, stability, and molecular interactions of PTEN can be either positively or negatively regulated; they can also impact its subcellular targeting (e.g., plasma membrane and nucleus). The review provides an extensive description of the different PTEN subdomains, as well as their structural and dynamic interactions, and details the distribution of the numerous PTMs along the molecule, as well as their biological impacts and the effector systems involved. Based on these insights, targeting effector systems that induce inhibitory post-translational modifications and/or enhancing PTEN-activating modifications appears to be a promising novel therapeutic strategy for PTEN-dependent tumors harboring at least one wild-type allele and for patients with PHTS.

In the case of nonsense mutations, Rafael Pulido’s Lab explored translational readthrough as an approach to restore PTEN function [8]. In this study, the authors demonstrate the importance of the identity of the premature termination codon (PTC) and nucleotide sequence upstream for efficient readthrough of the PTEN-L isoform. PTC might cause unstable transcripts, degraded through nonsense-mediated mRNA decay, and truncated/inactive/unstable proteins. They show that the readthrough of the L-PTEN termination codon in the coding sequence can be mediated by aminoglycosides, such as geneticin and gentamycin, enabling the incorporation of an amino acid in place of the termination codon. The limit of such an approach is aminoglycoside toxicity. Nevertheless, protein inducer Y-320 potentiates geneticin-induced PTEN readthrough. Thus, these combinations reveal potential alternative therapeutic approaches to explore for subgroups of patients with cancer or hamartoma syndrome bearing nonsense PTEN mutations.

Cellular plasticity underlying the epithelial–mesenchymal transition (EMT) is a key factor in carcinoma initiation. This process involves the loss of apical–basal polarity in epithelial cells, driven by decreased activity of junctional complexes, as well as the acquisition of mobility that enables cancer cell dissemination. Various EMT states coexist within tumors and also contribute to stem cell-like properties, dormancy, and resistance to chemotherapy. Olga Federova et al. discuss the regulation of PTEN by effectors of EMT [9]. These include ZEB1 and SNAI1/2 transcription factors, which impair PTEN transactivation, as well as a series of miRNAs that target PTEN transcripts. Interestingly, ZEB2 transcripts act as a PTEN ceRNA and thus might exert tumor-suppressive properties in some instances. The cross-talk between PTEN and signaling pathways involved in EMT, including integrins, TGFβ, NOTCH, and NF-kB cross-talk, and extrinsic factors such as cancer-associated fibroblast reactive oxygen species are also extensively documented.

Prostate cancers are characterized by a high incidence of PTEN mutations and deletions. The loss of both PTEN and p53 is considered a driver of metastatic progression in these cancers, which can be restrained by androgen deprivation therapy until the androgen receptor (AR) is amplified. In this context, Lloyd Trotman’s Lab developed engineered mouse models with conditional invalidation of both Pten and p53 which mimic human prostate cancer, including the metastatic process and acquired resistance to anti-androgen therapy. Using the RCaP cell line derived from this mouse model, as well as human prostate cancer cell lines and data mining, the authors demonstrate, in their contribution, that ectopic expression of FABP5 (fatty acid-binding protein 5) in advanced metastatic prostate cancer—associated with a poor prognosis—results from co-amplification of this genetic locus on chromosome 8q with c-MYC [10]. FABPs are involved in the uptake, metabolism, and nuclear delivery of specific fatty acids, wherein they activate nuclear receptors, such as PPARs, which promotes proliferation and survival. Among the cell lines investigated, only RCaP cells phenocopy incurable prostate cancer cells, i.e., resistance to AR antagonists and to taxanes (e.g., docetaxel) used in chemotherapy of prostate cancer. Interestingly, a family of FABP5 inhibitors decreased RCaP cell viability in vitro and inhibited tumor growth in vivo in nude mice. Thus, FABP5 inhibitors constitute a promising approach for the treatment of anti-androgen and docetaxel-insensitive prostate cancers—a feature related to Pten-defective tumors—which can be further addressed using an orthotopic xenograft of the Pten/Trp53-negative RCaP cells in immunocompetent mice as a preclinical model.

Pancreatic ductal adenocarcinoma (PDAC) is considered one of the cancers with the worst prognosis. Although genetic alterations in the “big four”—KRAS, p53, CDKN2A and SMAD4—are well documented, no efficient therapeutic strategy has emerged. Given the need to identify other effectors involved in pancreatic carcinogenesis in order to define more effective therapeutic targets, Laura M Porcza et al. focus in their review on non-genomic loss of PTEN, KDM6A and ARID1A tumor suppressors in PDAC [11]. They reveal that although PTEN mutation or deletion is relatively uncommon in PDAC, the loss of PTEN at the protein level is highly frequent, as evidenced by immunohistochemical studies (50% PDAC cases). Similarly, a decrease in protein levels of KDM6A and ARID1A are also observed. PTEN does not follow the “two-hit” inactivation hypothesis of tumor suppressors: PTEN dosage is a determining factor in the susceptibility of cancer development in a tissue-dependent manner; hence, its role in restraining PDAC remains underestimated. In connection with this, PTEN loss might strengthen the KRAS-induced PI3K pathway. Thus, PTEN, KDM6A and ARID1A and their effector systems should be considered as potential drivers in PDAC, broadening the scope of potential therapeutic targets.

As detailed above, many efforts have been directed towards developing therapeutic strategies restoring or strengthening PTEN activities to cure cancer and PHTS. However, other pathologies might benefit from transient PTEN inactivation. This has been demonstrated for the heart and brain following ischemia–reperfusion and for skin wound-healing using experimental mouse models. The last contribution of the Special Issue extends these observations to intestinal inflammation. An engineered mouse model with conditional Pten knockout targeted to intestinal epithelial cells shows increased proliferation leading to colonic hyperplasia [12]. Interestingly, these mice are less prone to chemically induced colitis due to enhanced intestinal barrier function linked to the strengthening of tight junctions. Furthermore, these mice recover faster after resolution of inflammation as a result of increased cell proliferation. In humans, ulcerative colitis is associated with an increased risk of developing colorectal cancer. Thus, paradoxically, localized transient downmodulation of PTEN using selective inhibitors could be an approach to promote healing of the colonic mucosa once the inflammation has subsided, which could potentially reduce the risk of developing cancer later on. The challenge of such a strategy lies in selectively targeting epithelial cells, as well as in determining the timing and duration of treatment to avoid any side effects.

## Concluding Remarks

The variety in the content of this collection provides an intelligible overview of the intricacy between the PTEN tumor suppressor and its numerous molecular partners and effector systems, as well as the many features of its biological activity. The complexity of this multifaceted tumor suppressor relies on fine-tuning at various levels and broad biological activities in distinct subcellular compartments, both related and unrelated to its phosphatase activity. A gap remains between molecular insights in PTEN function and translation in clinical practice. The contributions of this Special Issue pave the way towards informing the development of precision medicine targeting PTEN and/or its effector systems.

## Data Availability

No new data were created; data sharing is not applicable.
